# Straight intramedullary MultiLoc nails for displaced proximal humeral fractures: health status, radiographic results, clinical outcome, and complications

**DOI:** 10.1186/s12891-024-07656-y

**Published:** 2024-07-11

**Authors:** Wei Chen, Zhenhua Zhang, Chunhui Zhu, Zhiwen Song, Zhiyuan Liu

**Affiliations:** 1 Trauma Center, The Second People’s Hospital of Changzhou, Nanjing Medical University, Changzhou, 213003 China; 2 Department of Anesthesiology, The People’s Hospital of Danyang, Affiliated DanYang Hospital of Nantong University, Danyang, 212300 China; 3 Department of Spinal Surgery, The Third Affiliated Hospital of Soochow University, Changzhou, 213003 China; 4 Department of Orthopedics, Wujin Hospital Affiliated with Jiangsu University, Changzhou, 213003 China; 5 Department of Orthopedics, The Wujin Clinical College of Xuzhou Medical University, Changzhou, 213003 China

**Keywords:** Shoulder fractures, MultiLoc nails, Fracture fixation, Intramedullary, Treatment outcome, Postoperative complications

## Abstract

**Background:**

The treatment of the displaced proximal humerus fractures (PHF) still facing a lot of unsolved problems. The aim of this study was to evaluate the clinical effect of MultiLoc nails for the treatment of PHF and present outcomes of patients with different Neer’s classification and reduction quality.

**Methods:**

Adult patients with PHFs were recruited and treated with MultiLoc nail. Intraoperative data, radiographic and functional outcomes, as well as occurrence of postoperative complications were assessed.

**Results:**

48 patients met inclusion and exclusion criteria and were included in this study. The DASH Score were 32.2 ± 3.1 points at 12 months, and 37.3 ± 2.5 points at the final follow-up. The mean ASES score at 12 months and final follow-up were 74.4 ± 6.2 and 78.8 ± 5.1, respectively. The mean CM Score in all 48 patients reached 68 ± 6.4 points at the final follow-up, relative side related CM Score 75.2 ± 7.7% of contralateral extremity. The incidence rate of complications was 20.8%. Patients with fracture mal-union, adhesive capsulitis were observed but no secondary surgeries were performed. There was no significantly difference of DASH Score 12 months after surgery and at the last follow-up among patients with different Neer’s classification or reduction quality. However, functional outcomes such as ASES score and CM score were significantly influenced by severity of fracture and the quality of fracture reduction.

**Conclusions:**

Our study demonstrated that MultiLoc nails is well suited for proximal humeral fractures, with satisfactory health status recovery, good radiographic results, positive clinical outcomes and low rates of complications. The treatment for four part PHF still faces great challenges. Accurate fracture reduction was an important factor for good functional result.

## Introduction

Surgical treatment in proximal humerus fractures (PHF) is generally recommended for obvious displaced, unstable fracture patterns, but the ideal management of this fracture remains a topic of debate [1–3]. The goals of surgery are to obtain a better fracture reduction and a stable primary fixation to ensure rapid bone union and allow early postoperative mobilisation without prolonged immobilization [4, 5]. Frequently applied treatment options include locking plate fixation, intramedullary nailing and shoulder arthroplasty according to biological age and fracture patterns [6–8].

Among the many fixation methods available, intramedullary locking nails are commonly used, with several studies showing satisfactory clinical outcome results [6, 9]. The main drawbacks of the first and second generation of antegrade proximal humeral nailing include secondary dislocation of the fracture, screw loosening, chronic implant-related shoulder pain.

A higher risk of iatrogenic rotator cuff due to the entry point and a potential violation of the tendon of the long head of biceps brachii by the proximal anterior and posterior lateral locking-screws has also been reported in clinical cases [5–7, 10–12].

Recently, there are many studies comparing and evaluating the efficacy of intramedullary nails versus locking plates in the treatment of proximal humerus fractures. Interlocking Targon nail are more minimally invasive than locking plates and the intraoperative blood loss was much lower than that of the locking plate group [13]. Minghui Wang .et al found that TriGen straight nail has advantages over locking plates for OTA/AO type 11C1.1 and 11C3.1 proximal humerus anatomical neck fractures in terms of operation time and bleeding volume, with no significant difference in the number of complications [14]. A meta-analysis included 13 comparative studies with 958 patients indicates that locking plates and intramedullary nails have similar performance in terms of the functional scores and total complication rate [15].

As a third generation of intramedullary nail, straight MultiLoc Nail (DePuy Synthes, USA) could provide multi-planar distal fixation with angle-stable locking screws and an optional screw-in-screw concept. Further on, a calcar screw is introduced to increasing construct stability, which will play an important role in maintaining fracture reduction. Standard entry points in line with the humeral shaft axis could significantly reduce implant-related injuries to the adjacent anatomical structures in anatomical specimens [16]. Intramedullary nails demonstrated higher axial stiffness and smaller axial interfragmentary movements compared with locking plate designs in a biomechanical study [17]. Gomes GR .et al compared the proportions of complications and radiographic findings of Osteosynthesis of 2- and 3-part proximal humerus fractures with antegrade MultiLoc nail and locking plate. The nail group had less change in the postoperative cervicodiaphyseal angle [18]. MultiLoc nail with cement augmentation is a viable option for treating proximal humerus pathological fracture, providing less blood loss, rigid fixation and better pain relief [19]. However, superior clinical results have not been proven yet.

The purpose of the present study is to evaluate functional, radiological outcomes and potential complications of displaced PHFs using intramedullary MultiLocail nails.

We also analyse influence of Neer’s classification and the reduction quality on the functional outcome.

## Patients and methods

### Patients and interventions

This retrospective single-center study was conducted in a Level I trauma center at the Second People’s Hospital of Changzhou. Approval from the Ethics committee of the hospital was granted prior to initiation of the study(No.[2022]KY061-01).

**Fig. 1:**
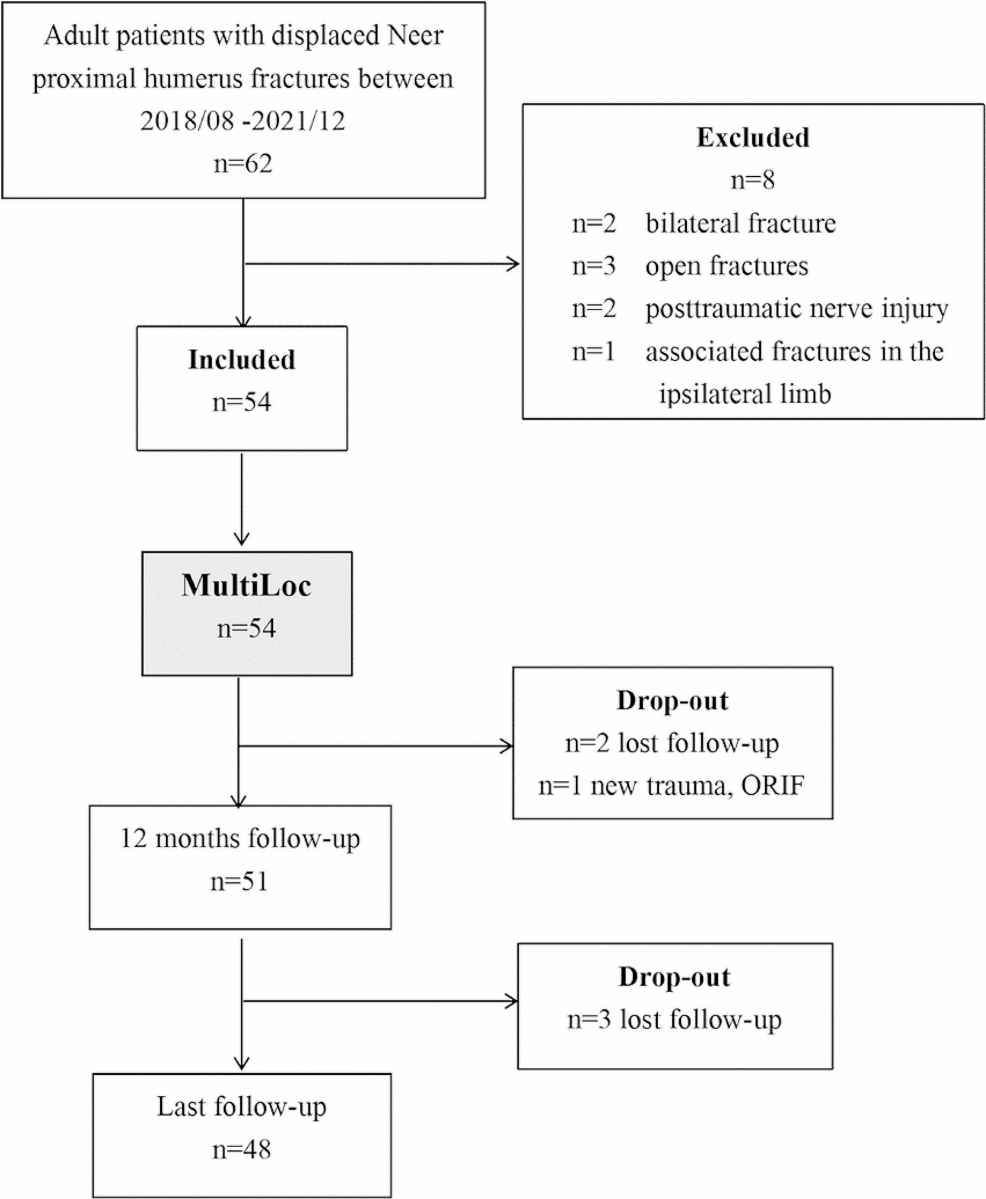
Flow diagram of patient enrolment and analysis. n, number; ORIF, Open reduction and internal fixation

Adult patients with displaced Neer proximal humerus fractures between August 2018 and December 2021 were recruited cumulatively and evaluated for eligibility. Patients with head-splitting fractures, bilateral fracture, pathological fractures, open fractures, posttraumatic nerve injury, associated fractures in the ipsilateral limb, previous surgery on the affected shoulder and comorbidities contraindicating surgery were excluded. Patients who were lost to follow-up or with new shoulder trauma on the ipsilateral side were excluded as well. Finally, 48 patients treated with an intramedullary MultiLoc Nail (DePuy Synthes, USA) were included in the study by a single senior surgeon(Fig. 1).

After X-ray with preliminary diagnose, 2D and 3D CT of injured proximal humerus with reconstructions was performed. Definitive decision about fracture type was made preoperatively. All fractures with technical possibility of reduction and fixation were indicated for reconstructive strategy. Operative treatment was exclusively performed by the study investigators, senior trauma surgeons experienced in the treatment of proximal humeral fractures.

### Surgical techniques

**Fig. 2:**
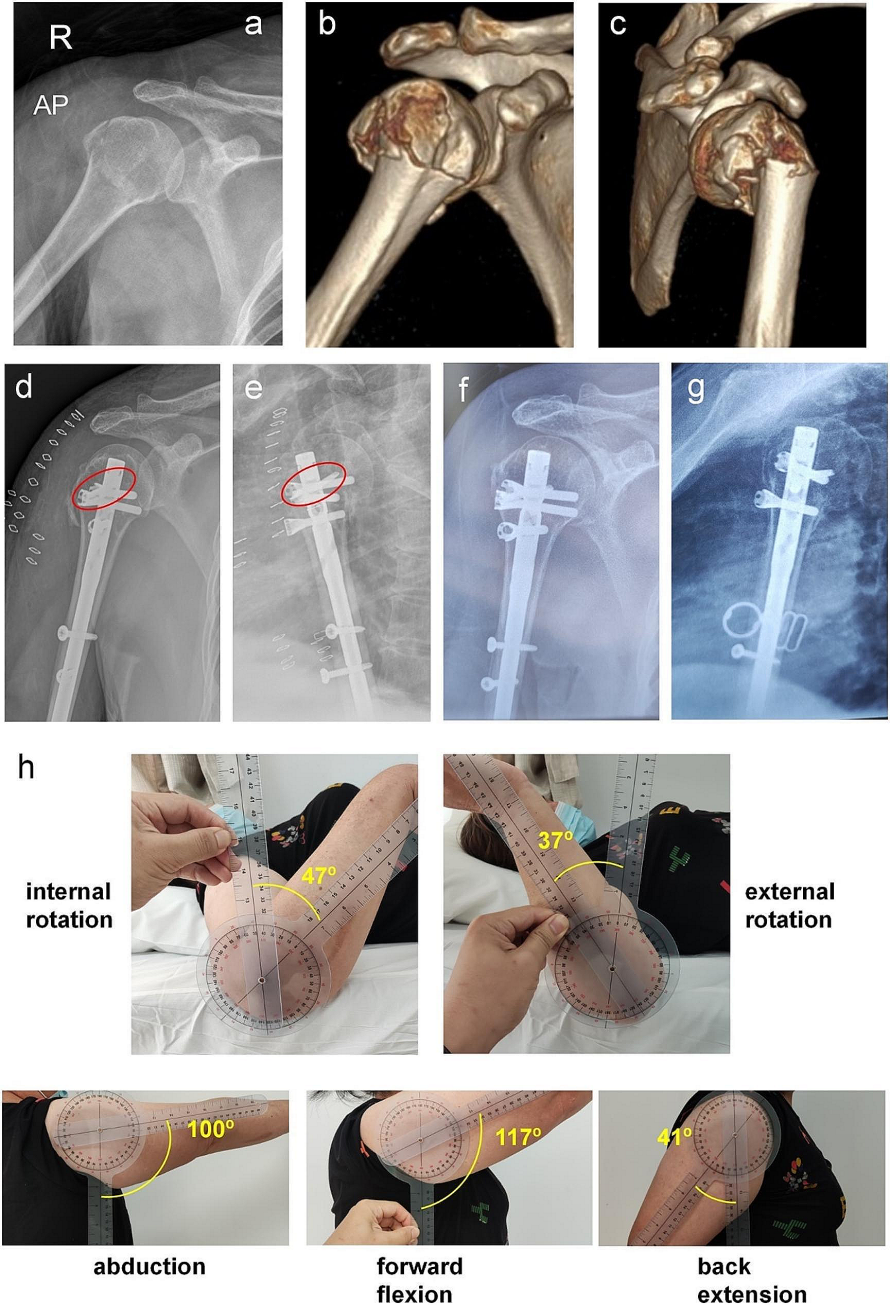
Female, 52 years old, three-part PHF. (**a**) Preop AP radiographs and (**b**, **c**) CT 3D reconstruction. Initial post-op (**d** and **e**) and 20 months post-op (**f** and **g**) showing fracture union without complication. (**h**) range of shoulder motion 20 months post-op

Surgery was performed with the patient under general anesthesia in beach chair position through an anterolateral deltoid split approach with arm in retroversion. Reduction of head fragments was performed individually according to the fracture patterns, with K-wires used in joystick technique, bone hooks and sutures. As for the 3- and/or 4-part fractures, non-absorbable suture -retracting technique was used to reduct the greater and lesser tuberosities to the humeral head first. Having confirmed adequate reduction, 2–3 cm long longitudinal incision into the supraspinatus tendon was performed. The entry point is situated at the apex of the humeral head, about 1.5 cm away from the cuff insertion onto the greater tuberosity. The position of the guiding wire was adjusted under fluoroscopic guidance. The medullary cavity was then opened with a hollow reamers. After that, the MultiLoc nail was inserted. Depth of insertion was adapted to the ideal position of proximal locking screws regarding fracture fragments. The number of proximal interlocking screws used was based on the fracture configuration and bone quality. Routinely, we made an attempt to insert the medial calcar screw. Screw-in-screw technique is applied to increase angular stability when there was poor bone quality(Fig. 2d and e). Briefly, after 4.5 mm MultiLoc locking screws was inserted proximally, specialized positioning sleeve could further locked with the MultiLoc screw by assembling with a T25 star shaped screwdriver. Then, a 3.5 mm interlocking screw was implanted through the head of the 4.5 mm locking screws. Distal locking by 2 screws was performed in all cases. Finally, Length of all locking screws was checked.

Whenever possible, the fragment control sutures were then inserted into the rotator cuff and tied to the suture holes on the interlocking screw heads to neutralize the pull of the rotator cuff and aid tuberosity fixation. After that, the rotator cuff was repaired. For patients with severe rotator cuff injury, we sutured the rotator cuff to the Multiloc screw hole. The deltoid fascia split was repaired. Finally, suture was closed by layers.

### Postoperative rehabilitation protocol

All patients prospectively underwent same post-surgical rehabilitation protocol that emphasized early passive and active-assisted motion exercises. The duration of protective immobilization with a sling was 4 weeks. The exercises begun on day one after surgery, immediately after diminishing of major postoperative pain. Maximum passive range of motion exercises was continued within the first 6 weeks. Then active movements started, with the aim of regaining full range of motion and normal strength as early as possible.

### Radiological assessment

Radiological evaluation took place immediately after surgery, after 3 months, and at the final follow up. Radiological outcomes were determined by two experienced surgeons.

The criteria for the quality of fracture reduction were adopted from previous studies, mainly depends on residual distance and angle between main fracture fragments. Under these considerations, patients were assigned in 3 groups according to Martin Kloub et al [20], rated as 1 = excellent, 2 = moderate and 3 = poor. Neck–shaft angle was also measured using the humeral shaft axis and line perpendicular to the articular segment or anatomic neck of the humerus.

Follow-up X-rays were used to identify union and all complications. Radiological union was defined as evidence of bridging external callous formation on radiographs, without shoulder pain or activity-related pain. If radiological union was not achieved 3 month after surgery, patients was asked to review every single month until the fracture healed. Complications assessed included mal-union, non-union, signs of avascular necrosis, resorption of the greater tuberosity and reduction/fixation failure.

### Clinical assessment

The primary outcome parameter was the Disabilities of the Shoulder, Arm and Hand (DASH) Score after 12 months and last follow-up. The main part of the DASH is a 30-item disability/ symptom scale ranging from 0 (no disability) to 100 (severest disability), concerning about the patient’s health status during the preceding week. Lower scores indicated better health status. Functional outcomes were also determined by using American Shoulder and Elbow Surgeons (ASES) shoulder score, the Constant–Murley Score (absolute), Side related relative CM score (relative), and range of motion (ROM) of shoulder joint after 12 months and/or final follow-up.

Postoperative complications including axillary nerve injury, infection of incisional wound, clinical signs and symptoms of subacromial impingement and/or rotator cuff tendon tears were recorded as well.

### Statistical analysis

Statistical analysis was performed with software Excel 2016, GraphPad Prism 7 and SPSS 22.0. The distributions of all numerical variables were evaluated for normality using the Shapiro-Wilk test. Data that satisfies normality were presented as the mean ± standard deviation. Those data that did not meet normality were presented as medians and quartiles.

Categorical data were represented by both a number and a percentage. Correlation between different Neer’s classification /reduction quality and DASH or functional scores(ASES score and CM score) were analyzed with one-way ANOVA or the Kruskal-Wallis test. *P* < 0.05 was regarded as statistical different.

## Results

The mean follow-up to latest functional assessment was 18.6 ± 3.2 months (range 12–24 months). The mean age was 58.6 ± 10.6 (range 40–75 years), with a male to female ratio of 20:28. According to Neer’s classification, 48 patients sustained 13 a two-part, 25 a three-part, and 10 a four-part fracture. Demographic characteristics are shown in Table 1.

**Table 1:** Demographic and surgical characteristics of patient cohort

**Age (years)**	
minimum- maximum, mean±SD	40-75(58.6±10.6)
**Sex** n(%)	
Male	20 (41.7%)
Female	28(58.3%)
**Localization** n**(**%)	
Left	28(58.3%)
Right	20(41.7%)
**Neer’s classification** n**(**%)	
2-part	13(27.1%)
3-part	25(52.1%)
4-part	10(20.8%)
**Time to operation**	
(days, mean±SD)	6.0±2.1
**Duration of surgery**	
(mins, mean±SD)	96.4±26.4
**hemorrhage**(ml, mean±SD)	59.0±24.7
**Follow-up** (months)	
minimum- maximum, mean±SD	12-24(18.6±3.2)

SD, Standard Deviation; n, number

### Surgical data

The mean delay time from injury to surgery was 6.0 ± 2.1 days. Average length of the operation were 96.4 ± 26.4 min, and average intraoperative hemorrhage were 59.0 ± 24.7 mL, as summarized in Table 1.

### Radiographic results

The mean intraoperative neck–shaft angle (NSA) was 129.9 ± 4.1º. In 19 cases was achieved excellent reduction, in 23 cases moderate, poor reduction was observed in 6 cases after osteosynthesis. All fractures achieved union.The mean radiographic fracture union time was 3.5 ± 0.7 months post-operation. The final NSA was 127.7 ± 5.1º.

### Clinical results

The DASH Score were 32.2 ± 3.1 points at 12 months, and 37.3 ± 2.5 points at the final follow-up. The mean American Shoulder and Elbow Surgeons(ASES) score at 12 months and final follow-up were 74.4 ± 6.2 and 78.8 ± 5.1, respectively. Average absolute CM Score in all 48 patients reached 68 ± 6.4 points at the final follow-up; relative side related CM Score 75.2 ± 7.7% of contralateral extremity. At last review, mean range of motion was forward flexion 135.9 ± 16.2º, abduction 125.6 ± 15.8º, external rotation 53.6 ± 8.2º, and internal rotation 42.3 ± 6.8º. Data of radiographic results, clinical outcome, and complication was summarized in Table 2. Documentation of one patient with three-part PHF was shown in Fig. 2.

**Table 2:** Radiographic results, clinical outcome, and complication

**Quality of reduction** ***n (%)***	
Excellent	19 (39.6%)
moderate	23 (47.9%)
Poor	6 (12.5%)
**Neck–shaft angle (NSA)** (º,mean±SD )	
intraoperative	129.9 ±4.1
final follow-up	127.7 ± 5.1
**Union time** (months, mean±SD)	3.5 ± 0.7
**DASH Score** (mean±SD)	
12month	32.2 ± 3.1
final follow-up	37.3 ± 2.5
**ASES score** (mean±SD)	
12month	74.4 ± 6.2
final follow-up	78.8 ± 5.1
**CM Score**	
absolute (mean±SD)	68.0 ± 6.4
relative (%)	75.2 ± 7.7%
**ROM (º**,mean±SD**)**	
forward flexion	135.9 ± 16.2
abduction	125.6 ± 15.8
external rotation	53.6 ± 8.2
internal rotation	42.3 ± 6.8
**Complication** n (%)	
mal-union	4 (8.3%)
adhesive capsulitis	8 (16.7%)

n, number; SD, Standard Deviation; DASH, disabilities of the arm, shoulder and hand; ASES, American shoulder and elbow surgeons; CM, Constant-Murley; ROM, range of motion

We further analyzed patient’s health status and functional outcomes according to Neer’s classification and quality of fracture reduction, as shown in Tables 3 and 4.

**Table 3:** Comparison of clinical results results according to Neer classifcations

	2-part (*n*=13)	3-part (*n*=25)	4-part (*n*=10)	F	*P*
**DASH score** (mean±SD)					
12 month	32.5 ± 3.7	32.1 ± 3.2	32.1 ± 2.4	0.056	0.946
final follow-up	37.9 ± 2.8	37.5 ± 2.2	35.9 ± 2.4	2.182	0.125
**ASES score** (mean±SD)					
12 month	77.3 ± 4.4	75.7 ± 5.0	67.2 ± 5.9	13.027	< 0.001
final follow-up	81.5 ± 3.6	79.9 ± 4.7	72.9 ± 3.2	13.736	< 0.001
**CM score**					
Absolute (mean±SD)	72.3 ± 3.8	68.6 ± 5.7	60.8 ± 4.9	14.629	< 0.001
Relative (%)	79.4± 5.7%	76.2 ± 7.0%	67.1 ± 5.7%	10.964	< 0.001

n, number; SD, Standard Deviation; DASH, disabilities of the arm, shoulder and hand; ASES, American shoulder and elbow surgeons; CM, Constant-Murley

**Table 4:** Comparison of clinical results according to reduction quality

	excellent (*n*=19 )	moderate (*n*=23)	poor (*n*= 6)	F	*P*
**DASH score** (mean±SD)					
12 month	32.1 ±2.6	32.8 ± 3.5	30.5 ± 2.9	1.138	0.278
final follow-up	37.5 ± 2.3	37.3 ± 2.6	35.2 ± 1.7	2.689	0.079
**ASES score** (mean±SD)					
12 month	77.5 ± 5.8	74.3 ± 4.0	64.5 ± 4.0	16.566	< 0.001
final follow-up	81.4 ± 4.8	78.6 ± 4.2	72 ± 2.4	11.03	< 0.001
**CM score**					
Absolute (mean±SD)	71 ± 4.6	67.2 ± 7.0	61.5 ± 3.4	6.512	< 0.001
Relative (%)	78.9 ± 5.6%	74.2 ± 8.2%	67.4 ± 4.1%	6.909	< 0.001

n, number; SD, Standard Deviation; DASH, disabilities of the arm, shoulder and hand; ASES, American shoulder and elbow surgeons; CM, Constant-Murley

We did not find any statistical relationship between DASH Score and Neer classifcations ( *p* = 0.946, at 12 months and *p* = 0.125, at the last follow-up) or reduction quality ( *p* = 0.278, at 12 months and *p* = 0.079, at the last follow-up). However, at 12 months, the mean ASES score in four-part PHF was much lower (67.2 ± 5.9) than these in two-part PHF (77.3 ± 4.4) and three-part (75.7 ± 5.0) ( *p* < 0.01). Moreover, worse functional outcomes were observed in the four-part (ASES score 67.2 ± 5.9 and absolute CM score 60.8 ± 4.9, relative CM score 67.1 ± 5.7%) compared to the two-part (ASES score 81.5 ± 3.6 and absolute CM score 72.3 ± 3.8, relative CM score 79.4 ± 5.7%) and three-part groups (ASES score 79.9 ± 4.7 and absolute CM score 68.6 ± 5.7, relative CM score 76.2 ± 7.0%) at the last follow-up. All *p* < 0.001.

ASES scores in the group of excellent reduction achieved 77.5 ± 5.8 at 12 months and 81.4 ± 4.8 at the last follow-up comparing with the group of moderate (74.3 ± 4.0, 78.6 ± 4.2) and poor reduction ( 64.5 ± 4.0, 72 ± 2.4) ( *p* < 0.001, *p* < 0.001). At the final follow-up, absolute/relative CM score in the group of poor reduction was 61.5 ± 3.4 / 67.4 ± 4.1% and significantly lower than these in the group of moderate and excellent reduction ( *p* = 0.003 and *P* = 0.002).

### Complications

Surgical complications including axillary nerve injury, infection of incisional wound and rotator cuff tear were not found. No radiographic evidences of screw cut-out or backout, failure of fixation, nonunion or avascular necrosis of the humeral head was identified at the final follow-up. The incidence rate of complications was 20.8%. Fracture mal-union occurred in 4 cases (8.3%), adhesive capsulitis in 8 cases (16.7%), but no secondary surgeries were performed.

## Discussion

The purpose of this retrospective **study** was to evaluate the efficacy and outcome after treatment of proximal humeral fractures with third-generation straight intramedullary Multiloc nails. The major findings of this **study** is that MultiLoc nail is well suited for the treatment of PHF with satisfactory recovering of health status, as well as good radiological and clinical outcomes, low rates of complications. Our study also yielded further results. First, four-part type PHF resulted in worse functional outcome. Second, if better reduction quality achieved, clinical outcome dramatically raised.

Owing to its biomechanical advantages, locked intramedullary nailing is emerging as a preferred technique in managing displaced proximal humerus fractures in appropriately selected patients in clinical practice [21, 22]. Several studies demonstrated good functional outcomes for intramedullary nails, similar to locking plates, in the treatment of displaced fractures [23–25].

As a third-generation straight intramedullary nails, the MultiLoc^®^ humeral nail is an implant with locked properties that offers numerous modular proximal locking options, compared with previous generations of intramedullary nails [6], MultiLoc nail configurated with screw-in-screw and calcar screw could support the humeral head fragment in both axial and rotational direction to prevent varus collapse and reduce the risk of secondary redislocation in clinical use [26]. Additionally, MultiLoc nail with a appropriate endcap significantly reduced varus-displacing forces to the humeral head and provided increased stability [27]. All of these design of MultiLoc nail could be advantageous in case of poor fixation, due to osteoporosis or a complex fracture pattern, as shown in Table 5.

**Table 5:** Characteristics of different generations of antegrade humeral nail designs

**“First-generation” nailing**	**“Second-generation” nailing**	**“Third-generation” MultiLoc Nail**
(Rush nail)	(the Polarus nail, the Telegraph nail, Targon PH and so on)	
		**General Advantages**
**1** Fail to provide adequate fixation of displaced fragments	**Advantages**	**1** more secure locking mechanisms for proximal screw fixation to allow fixed angular stable constructs and lead to reliable stability
**2** No rotational control.	**1** a spiral array of interlocking screws	**2** Straight nails reduce rotator cuff injuries
**3** Fail to neutralize the deforming forces and often lead to malunion or nonunion.	**2** radiolucent targeting guide	**3** reduce acromial contact
**4** acromial contact and requiring a second procedure for removal.	**3** axillary nerve window to avoid nerve injury	**4** Reduced risk of nerve damage
	**4** calibrated drills and drill guides	**Unique Advantages**
	**5** better rotational control	**1** Multi-dimensional fixation of proximal and distal of humerus
	**The major disadvantage**	**2** target the posteromedial region with strong bonemineral density
	poor angular stablity and engaged only the osteoporotic bone lead to loss of fixation or screw backout	**3** Screw-in-screw concept
		**4** additional calcar screw
		**5** polythylene liner
		**6** screw head suture holes to enable rotator cuff attachment

Recently, MultiLoc nail is already considered as a reliable technology for the treatment of more complex fractures of proximal humerus. Several clinical trials reported good clinical and radiographic outcomes for MultiLoc^®^ nail, even in the elderly patients. Toon, et al. investigated the clinical data of 22 consecutive patients with PHF underwent MultiLoc nail treatment. Good early outcomes and low rates of complications demonstrated that MultiLoc nail is well suited for Neer’s two- and three-part proximal humeral fractures [28]. In elderly patients, osteoporosis makes internal fixation problematic and frequently contributes to failed fixation and poor clinical results. In a prospective randomized controlled clinical trial, MultiLoc nail as well as Locked plating with screw-tip-augmentation achieve satisfying functional outcomes in 2-part surgical neck type fractures of the proximal humerus in an elderly population at two years of follow-up [29]. Yaiza, et al. found proper operative technique with a MultiLoc nail result in good functional results and good HRQol with a low complication rate in elderly patients aged 80 y.o. or older presenting a two-part or three-part PHFs [30]. In our study, treatment of the PHF including three and four part by intramedullary MultiLoc nail provided good DASH score as high as 37.3 ± 2.5, representing a satisfactory health status after surgry. Positive radiographic and functional outcomes and low rates of postoperative complications were also achieved. There was no ununion, avascular necrosis and secondary surgeries at the final follow-up.

The treatment strategy of displaced 4-part proximal humeral fractures is still in the focus of the interest. Conservative management, locked PHILOS plate, hemi-arthroplasty, or reverse total shoulder arthroplasty, including intramedullary nails, were all chosen as possible therapies [31–34]. However, no intervention was considered as the most appropriate for their management currently. Greenberg, et al.evaluated the radiographic and clinical outcomes of 23 patients with low-energy, osteoporotic, 4-part proximal humeral fractures underwent fixation via Targon intramedullary nails [35]. They found intramedullary nails can successfully be used by experienced surgeons in fixation of 4-part PHFs with osteoporosis. Toon, et al. found the outcome for four-part fractures after MultiLoc nail treatment were inferior to two or three-part fractures in a single-institution prospective cohort study [28]. In another prospective monocentric cohort study, 40 patients with displaced four-part proximal humeral fractures were treated with MultiLoc nail. Complications like head necrosis and failure of fixation were not rare at the final follow-up [36]. In this study, we evaluated 10 patients with 4-part fracture in average age 55.9 ± 12.7, with average ASES score 72.9 ± 3.2, absolute CM score 60.8 ± 4.9 and relative CM score 67.1 ± 5.7% at the final follow-up, significantly lower than 2-part or 3-part fractures. These results were similar to previous researches.

In addition to the classification, appropriate reduction of fracture fragments also have a great impact on clinical result. Influence of reduction quality on the functional result was already observed and most studies report worsening of outcome in malreduced or malunited fractures [37–39]. Martin, et al. reassessed long term results of 137 patients with three or four-part fractures of the humeral head treated by intramedullary nailing. Long term results confirmed nailing as appropriate treatment strategy for all types of humeral head fractures with limitation of excellent reduction in every age group [20]. Similarly, better functional result and lower rates of complications in patient treated with MultiLoc nail depending on surgical technique, especially accurate reduction [29, 36]. We compared ASES score and CM score among patients with different reduction quality. Better quality of fracture reduction corresponds to higher ASES score while poor reduction led to lower CM score, indicating that functional outcome is strongly influenced by the grade of achieved reduction.

Outcomes after MultiLoc nail magement also involved complications after surgical operation. In 48 patients there were together 12 complications until final follow-up.

Implant related complications like screw cut-out or back-out, loss of reduction were not found in our study group, similar to previous studies [40]. Martin, et al. reported complete or partial head necrosis, resorption of the greater tuberosity in 40% in patients with displaced four-part proximal after intramedullary nails treatment [36]. However, these complications was not occurred in our study cohort. This may be explained by lower proportion of four-part fractures and higher reduction quality of fractures. Nevertheless, 4 cases (17.1%) of mal-union was happened, mainly found in patients with poor reduction quality. Adhesive capsulitis was found in eight cases, which might be attribute to primary or implant mal-position induced rotator cuff injury or negative rehabilitation.

There are several limitations in our study. The first limitation could be different length of follow-up time, which varies from 12 to 24 months, might influence clinical outcomes. Fortunately, we have got DASH score and ASES score 12months after surgical operation. Secondly, the results were based on patients treated by the same surgeon in our study. This may affect the further generalizability of the conclusion. On the other hand, data bias is reduced when we further investigate the outcome-related factors. Other limitations of this study were a retrospective design, small number of cases and only a short-term outcome.

The strength of this study is the use of multiple objective and subjective clinical-functional outcomes, especially DASH score concerning patient’s health status. Further studies with larger patient numbers, prospective randomized controlled clinical trial, even multicenter cohort study, are still needed to determine the role and efficiency of MultiLoc nail for proximal humeral fracture fractures.

## Conclusions

In conclusion, MultiLoc nail resulted in a satisfactory clinical outcome for the treatment of proximal humeral fractures, with a reasonable low complication rate. The technique is suitable to help patient regain health status after operation. Optimum management choice of four-part fractures still facing a big challenge. Appropriate reduction of the fracture during the operation is a key for a good functional result.

## Data Availability

The datasets used and/or analyzed during the current study available from the corresponding author on reasonable request.
